# A Review of Introducing Issues with Opposing Viewpoints: Animal Rights

**DOI:** 10.3390/ani1030256

**Published:** 2011-07-06

**Authors:** Lee J. Markowitz

**Affiliations:** Department of Psychology, La Roche College, Pittsburgh, PA 15237, USA; E-Mails: lee.markowitz@laroche.edu; lee.markowitz@yahoo.com

Given the volatile nature of animal rights issues and the extensive array of writings on the topic, one might expect several introductory anthologies to be available. The only anthologies in print, however, are scholarly tomes (listed below) geared towards more advanced readers. Fortunately, Lauri S. Friedman, author of dozens of anthologies on controversial topics such as gun control, national security, terrorism, fast food, sexually transmitted diseases, and many other topics, fills this void well with her volume titled *Introducing Issues with Opposing Viewpoints: Animal Rights*. She has included articles by influential authors in a diverse range of lay outlets such as *The Wall Street Journal, Slate, Guardian, Christianity Today, Food & Wine*, among others. Below, I describe the contents of the book, its strengths and weaknesses, and how educators might use the book in classroom settings.

The book is divided into three sections. The first section is titled “Should animals have rights?” and has four articles by authors supporting (Richard Ryder, Patrick Battuello, Josephine Donovan, and Justin Goodman) and four by authors opposing (William Saletan, Wesley Smith, Charles Colson and Anne Morse, and Michael Conn) animal rights. The supporters base their arguments on animal sentience and the degree of suffering caused by industries using animals. The focus in two of the papers opposing animal rights is on morally relevant qualities that humans allegedly have and animals allegedly lack: advanced cognitive and emotional abilities (Saletan) or souls (Colson and Morse). The other two opponents focus on potential costs of establishing rights for animals to industries using animals (Smith and Conn). Overall, these eight readings provide a good overview of the most frequent arguments in favor of and opposing animal rights and help to lay the foundation for the literature on more narrow issues covered in the next two sections.

The second section is titled “How should animals be treated,” although it focuses solely on the ethics of our food choices. Jonathan Safran Foer argues against eating animals by focusing on the mental similarities between dogs (an animal species most Americans would never eat) and pigs (whom most Americans do eat). Gary Francione advocates a vegan diet because of how inhumane factory farming is and because there is no nutritional need to eat meat. In contrast, Christine Lennon argues that locally raised meat is a healthy and ethical choice. Finally, Natasha Mann claims that vegan diets are impractical and can be dangerous to health. Although the articles in this section provide a good representation of the common arguments for and against meat consumption, I was disappointed that the authors rely only on personal anecdotes rather than citing the large body of systematic research on the health effects of vegan diets [1].

In the final section of the book, George Poste, Robert Winston, and John Illman argue that research on animals is necessary to advance human health by citing instances where such research facilitated medical advances. In contrast, Peter Tatchell cites instances where generalizing results from animal research to humans has led to costly errors and concludes that alternatives to animal research are more useful. Although the pros and cons for human welfare of animal research are interesting to discuss, the topic rests on a speciesist assumption that none of these authors addresses: human life is more important than nonhuman animal life. Alistair Currie, in contrast, questions this assumption. She argues against animal research because “means don't justify ends” (p. 113). Thus, readers of this section will be introduced to arguments on the costs and benefits of nonhuman animal research and will be encouraged to question speciesist assumptions underlying the research. After this section, Freidman includes eighteen pages of additional resources: statistics on animal use in various industries, websites for relevant organizations, and citations for further readings.

Friedman's book has several noteworthy strengths. First, for a 144-page book, its breadth of coverage is excellent. On several contentious topics, readers are introduced to the main arguments for and against animal rights. A second strength is that the papers are highly readable—even for individuals new to the animal rights movement. Third, in brief commentaries, Friedman provides helpful summaries of the main arguments of each paper and questions to consider. Finally, because the book is an anthology, the authors express themselves more engagingly and passionately than the typical content of introductory textbooks, which tend to be more neutral in tone. Anyone interested in animal rights should find the book captivating.

Despite these strengths, I was disappointed that there were no writings by Peter Singer or Tom Regan, who are widely considered the two founders of the modern animal rights movement. Because of these omissions, the reader is not introduced to the natural rights (Regan) or utilitarian (Singer) animal rights perspectives. I do not view this omission as devastating, though, and believe introductory readers will benefit from reading Friedman's volume. The book should be useful to anyone interested in animal rights in the general public and to course instructors. However, instructors may want to supplement the readings from this anthology with selections from more advanced anthologies [2-4]. Given the dearth of introductory level anthologies on the topic, Friedman's book fills a critical void and paves a path for future, more exhaustive introductory-level anthologies.
